# Melhora no Consumo Máximo de Oxigênio e na Ventilação após Tratamento com Sacubitril-Valsartana

**DOI:** 10.36660/abc.20190443

**Published:** 2020-10-09

**Authors:** António Valentim Gonçalves, Tiago Pereira-da-Silva, Ana Galrinho, Pedro Rio, Rui Soares, Joana Feliciano, Rita Ilhão Moreira, Sofia Silva, Sandra Alves, Eunice Capilé, Rui Cruz Ferreira

**Affiliations:** 1 Hospital de Santa Marta Lisboa Portugal Hospital de Santa Marta, Lisboa – Portugal

**Keywords:** Insuficiência Cardíaca, Consumo de Oxigênio, Fração de Ejeção Ventricular, Ventilação Pulmonar, Sacubitril-Valsartana, Anti-Hipertensivos

## Abstract

**Fundamento:**

O tratamento com sacubitril-valsartana teve seu benefício prognóstico confirmado no ensaio PARADIGM-HF. No entanto, dados sobre alterações no teste de esforço cardiopulmonar (TECP) com o uso de sacubitril-valsartana são escassos.

**Objetivo:**

O objetivo deste estudo foi comparar os parâmetros do TECP antes e depois do tratamento com sacubitril-valsartana.

**Métodos:**

Avaliação prospectiva de pacientes com insuficiência cardíaca (IC) crônica e fração de ejeção do ventrículo esquerdo ≤40%, mesmo sob terapia padrão otimizada, que iniciaram tratamento com sacubitril-valsartana, sem expectativa de tratamentos adicionais para a IC. Os dados do TECP foram coletados na semana anterior e 6 meses depois do tratamento com sacubitril-valsartana. Diferenças estatísticas com valor p <0,05 foram consideradas significativas.

**Resultados:**

De 42 pacientes, 35 (83,3%) completaram o seguimento de 6 meses, uma vez que 2 (4,8%) morreram e 5 (11,9%) interromperam o tratamento devido a eventos adversos. A média de idade foi de 58,6±11,1 anos. A classe NYHA (classificação da New York Heart Association) melhorou em 26 (74,3%) pacientes. O consumo máximo de oxigênio (VO_2_max) (14,4 vs. 18,3 ml/kg/min, p<0,001), a inclinação VE/VCO_2_ (36,7 vs. 31,1, p<0,001) e a duração do exercício (487,8 vs. 640,3 s, p<0,001) também melhoraram com o uso de sacubitril-valsartana. O benefício foi mantido mesmo com a dose de 24/26 mg (13,5 vs. 19,2 ml/kg/min, p=0,018) de sacubitril-valsartana, desde que esta tenha sido a maior dose tolerada.

**Conclusões:**

O tratamento com sacubitril-valsartana está associado a uma melhora acentuada do VO_2_max, da inclinação VE/VCO_2_ e da duração do exercício no TECP. (Arq Bras Cardiol. 2020; [online].ahead print, PP.0-0)

## Introdução

O prognóstico de pacientes com insuficiência cardíaca (IC) mudou consideravelmente após a publicação de ensaios fundamentais (1987 – CONSENSUS,^1^ 1999 – CIBIS-II^2^ e RALES^3^ ), que demonstraram o benefício do uso de antagonistas neuro-hormonais [inibidores da enzima conversora da angiotensina (IECA), betabloqueadores (BB) e antagonistas da aldosterona (AA), respectivamente] na melhora da sobrevida de pacientes com IC e na redução da fração de ejeção.

Vinte e sete anos depois do ensaio CONSENSUS, o ensaio PARADIGM-HF mostrou que o uso de sacubitril-valsartana, uma combinação de inibidor da neprilisina e bloqueador do receptor da angiotensina II (BRA), poderia reduzir a hospitalização por IC e a mortalidade cardiovascular em 20% em comparação com o Enalapril.^4^

Como resultado, o sacubitril-valsartana recebeu uma recomendação de Classe I, nível de evidência B, como substituto do IECA em pacientes ambulatoriais com IC e fração de ejeção reduzida (ICFER) que permanecem sintomáticos mesmo sob tratamento ideal com IECA (ou BRA se o IECA não for tolerado), BB e AA.^5^ Contudo, o uso de sacubitril-valsartana não tem sido tão alto quanto esperado.^6^

Os objetivos do tratamento para pacientes com IC não são apenas prevenir a internação e reduzir a mortalidade, mas também melhorar o estado clínico e a capacidade funcional. O teste de esforço cardiopulmonar (TECP) é um forte preditor de mortalidade em pacientes com IC. Ele é utilizado como critério padrão para avaliar a necessidade de transplante cardíaco eletivo,^7^ sendo o consumo máximo de O_2_ (VO_2_max) e a relação entre ventilação e produção de CO_2_ (inclinação VE/VCO_2_) as ferramentas de avaliação de risco mais adotadas.^8^

O volume de informação tem aumentado recentemente, já que alguns ensaios demonstraram melhora sintomática e funcional significativa após o início do tratamento com sacubitril-valsartana.^9 - 12^ Entretanto, o impacto na capacidade funcional requer pesquisas adicionais, pois a maioria dos ensaios tinha delineamento retrospectivo e, até onde sabemos, apenas um estudo prospectivo apresenta as alterações de parâmetros do TECP após o tratamento com sacubitril-valsartana.^13^

O objetivo deste estudo foi analisar prospectivamente a eficácia do tratamento com sacubitril-valsartana em uma coorte de pacientes com IC crônica sob terapia padrão otimizada, comparando os dados do TECP antes e depois do tratamento.

## Métodos

A pesquisa foi conduzida em conformidade com os princípios estabelecidos na Declaração de Helsinque. O comitê de ética institucional e a Comissão Nacional de Proteção de Dados (CNPD, número de autorização 5962) aprovaram o protocolo do estudo.

Todos os pacientes forneceram consentimento livre e esclarecido por escrito.

### População de pacientes

O estudo apresenta uma análise prospectiva de único centro realizada entre outubro de 2017 e junho de 2018.

Durante esse período, todos os pacientes ambulatoriais sob terapia padrão otimizada para IC crônica, com fração de ejeção do ventrículo esquerdo ≤40% e classe ≥ II na classificação da New York Heart Association (NYHA) foram orientados a iniciar o tratamento com sacubitril-valsartana de acordo com as diretrizes vigentes.^5^

### Definição de IC crônica sob terapia padrão otimizada

A terapia padrão otimizada para IC crônica foi definida como mais de seis meses de tratamento com a dose máxima tolerada de um IECA ou BRA, conforme apropriado, um BB e um AA. O cardioversor desfibrilador implantável (CDI) e/ou a terapia de ressincronização cardíaca (TRC) podem ser utilizados se indicados pelas diretrizes vigentes e se o paciente tiver sido adequadamente tratado de acordo com os padrões aplicáveis para doença coronariana e insuficiência mitral (IM)^5^ e não haja expectativa de alteração do tratamento adicional da IC nos 6 meses seguintes. Foram excluídos os pacientes que iniciaram um programa de exercícios nos 3 meses anteriores ou durante o tratamento com sacubitril-valsartana.

### Protocolo do estudo

Todos os pacientes forneceram consentimento livre e esclarecido por escrito. Em seguida, foram obtidos dados clínicos, laboratoriais, de ecocardiograma transtorácico (ETT) e de TECP na semana anterior ao início do tratamento com sacubitril-valsartana.

Um período de eliminação ( *washout* ) de 36 horas permitiu a alteração de um IECA para o sacubitril-valsartana. O tratamento com sacubitril-valsartana foi preferencialmente iniciado com a dose de 49/51 mg duas vezes ao dia ou 24/26 mg duas vezes ao dia em pacientes com dose <10 mg/dia de Enalapril ou equivalente. Foram feitas tentativas de duplicar a dose a cada 2 a 4 semanas para atingir a dose-alvo de manutenção de 97/103 mg duas vezes ao dia, exceto em pacientes com pressão sistólica <100 mmHg, hipotensão sintomática, hiperpotassemia > 5,5 mEq/L ou diminuição da taxa de filtração glomerular (TFG) para menos de 60 ml/min, avaliada pela equação de Cockcroft-Gault.

Todos os pacientes foram acompanhados por seis meses a partir da data de conclusão dos exames e os dados clínicos, laboratoriais, de ETT e de TECP foram coletados novamente após seis meses de tratamento com sacubitril-valsartana.

O apêndice complementar fornece informações relativas a todos os dados levantados.

### Teste de esforço cardiopulmonar

Um TECP máximo ou sintoma-limitado foi realizado em esteira utilizando o protocolo de Bruce modificado (esteira ergométrica GE Marquette Série 2000). A análise de gases foi precedida pela calibração do equipamento. A ventilação minuto, o consumo de oxigênio e a produção de dióxido de carbono foram obtidos respiração a respiração, por meio de um analisador de gases SensorMedics Vmax 229. O VO_2_max foi definido como a maior média de 30 segundos alcançada durante o exercício e foi normalizado para o índice de massa corporal.^14^ O limiar anaeróbio (LA) foi determinado pela combinação de métodos padrão (inclinação V preferencialmente e equivalentes ventilatórios). A inclinação VE/VCO_2_ foi calculada por regressão linear por mínimos quadrados, com base nos dados adquiridos ao longo de todo o exercício. Os pacientes foram incentivados a realizar o exercício até que a razão de troca respiratória (RTR) fosse ≥1,10.

### Análise estatística

As características iniciais são expressas em frequências (porcentagens) para variáveis categóricas e como médias e desvios padrão para variáveis contínuas. Todas as análises comparam os parâmetros dos pacientes no início e seis meses depois do tratamento com sacubitril-valsartana.

A distribuição normal das variáveis contínuas foi verificada pelo teste de Kolmogorov-Smirnov. O teste t para amostras pareadas comparou as variáveis antes e depois do tratamento com sacubitril-valsartana. Diferenças estatísticas com valor p <0,05 foram consideradas significativas. Os dados foram analisados no programa Statistical Package for the Social Science para Windows, versão 24.0 (SPSS Inc, Chicago, IL).

## Resultados

### Visão geral da população estudada

Um total de 42 pacientes participaram do estudo. Destes, 35 (83,3%) completaram o seguimento de seis meses com sacubitril-valsartana, já que 2 (4,8%) pacientes morreram (1 com hemorragia intracraniana pós-trauma e 1 com morte súbita cardíaca) e 5 (11,9%) interromperam o tratamento devido a eventos adversos (2 com insuficiência renal aguda reversível e 3 com hipotensão sintomática com a menor dose de sacubitril-valsartana). Não houve perda de seguimento durante os seis meses.

A Tabela 1 apresenta as características iniciais dos 35 pacientes que completaram o seguimento de seis meses com sacubitril-valsartana. A média de idade foi de 58,6±11,1 anos, com 29 (82,9%) pacientes do sexo masculino e etiologia isquêmica em 15 (42,9%) participantes.

**Tabela 1:** – Características iniciais da população do estudo (n=35)

Características	n (%)
Média de idade (anos)	58,60 ± 11,14
Etiologia isquêmica	15 (42,9%)
Sexo masculino	29 (82,9%)
NYHA ≥ III	18 (51,4%)
Média do índice de massa corporal (kg/m^2^)	28,09 ± 3,77
Hospitalização por insuficiência cardíaca no ano anterior	15 (42,9%)
Média de PNB (pg/ml)	375,30 ± 342,19
Fumante	7 (20,0%)
Hipertensão anterior	25 (71,4%)
Dislipidemia	25 (71,4%)
Diabetes mellitus	11 (31,4%)
Doença arterial periférica	4 (11,4%)
Histórico familiar de insuficiência cardíaca	1 (2,9%)
Fibrilação atrial	14 (40%)
Doença renal crônica	2 (5,7%)
Doença hepática crônica	0 (0,0%)
Inibidores da enzima conversora de angiotensina	29 (82,9%)
Bloqueador do receptor de angiotensina II	6 (17,1%)
Betabloqueadores	35 (100,0%)
Antagonista da aldosterona	33 (94,3%)
Ivabradina	13 (37,1%)
Digoxina	9 (25,7%)
Cardioversor desfibrilador implantável	30 (85,6%)
Terapia de ressincronização cardíaca (TRC-D)	7 (20%)
Reparo percutâneo da valva mitral com MitraClip®	3 (8,6%)

*NYHA: New York Heart Association; PNB: peptídeo natriurético tipo B.*

Esses pacientes eram altamente sintomáticos, como comprovado pela classe NYHA ≥ III em 51,4% deles e pelos 42,9% de hospitalizações por piora da IC no ano anterior ao tratamento com sacubitril-valsartana. Todos os pacientes usavam um IECA ou BRA associado a um BB e 94,3% tomavam um AA. O CDI já estava implantado em 30 (85,6%) pacientes, dos quais 7 (20,0%) tinham o sistema TRC-D. Três (8,6%) pacientes haviam sido previamente submetidos a um reparo percutâneo da valva mitral por meio do sistema de MitraClip®.

### Dose de sacubitril-valsartana

O tratamento com sacubitril-valsartana foi iniciado com doses de 24/26 mg duas vezes ao dia em 18 (51,4%) pacientes e 49/51 mg duas vezes ao dia em 17 (48,6%) pacientes. Aos seis meses, a dose de 24/26 mg duas vezes ao dia era administrada a 10 (28,6%) pacientes, 49/51 mg duas vezes ao dia a 11 (31,4%) e 97/103 mg duas vezes ao dia a 14 (40,0%).

Não foram encontradas alterações significativas quanto à dose expressa em porcentagem da dose-alvo de BB (68,8 ± 28,6% vs. 70,6 ± 28,0%, p=0,278) e AA (51,6 ± 19,0% vs. 53,2% ± 24,4%, p=0,352) ou à dose de diurético de alça expressa em equivalentes de furosemida (43,6 ± 27,6% vs. 39,1 ± 26,5%, p=0,191) no início e após seis meses do tratamento com sacubitril-valsartana.

### Avaliação clínica

Os 35 pacientes que completaram os seis meses de tratamento com sacubitril-valsartana apresentaram uma melhora relevante na classe NYHA, já que apenas 9 (25,7%) deles permaneceram na mesma classe, enquanto 24 (68,6%) melhoraram uma classe NYHA e 2 (5,7%) melhoraram duas classes. Nenhum paciente teve piora da classe NYHA durante os seis meses de tratamento com sacubitril-valsartana e apenas 3 (8,6%) permaneceram na classe III.

### Avaliação do ecocardiograma transtorácico

A Tabela 2 apresenta os resultados da análise do ETT. As dimensões do ventrículo esquerdo (VE) e os volumes atriais foram significativamente menores aos seis meses de tratamento. A excursão sistólica do anel tricúspide não apresentou diferenças significativa, independentemente da presença de diminuição na pressão sistólica da artéria pulmonar aos 6 meses de terapia. A fração de ejeção do ventrículo esquerdo teve elevação média absoluta de 5,9%.

**Tabela 2:** – Dados ecocardiográficos antes e seis meses depois do tratamento com sacubitril-valsartana

		Tempo 0	6 meses	p
**Dados Ecocardiográficos**				
Diâmetro diastólico final do ventrículo esquerdo (mm)		71,3 ± 8,4	66,9 ± 7,6	0,001
Diâmetro sistólico final do ventrículo esquerdo (mm)		57,8 ± 9,4	53,1 ± 9,3	0,002
Septo interventricular (mm)		9,6 ± 1,7	9,9 ± 1,9	0,280
Fração de ejeção do ventrículo esquerdo (%)		29,3 ± 6,4	35,17 ± 8,6	0,001
Volume do átrio esquerdo (ml/m^2^)		51,5 ± 22,6	43,7 ± 15,8	0,004
Volume do átrio direito (ml/m^2^)		33,1 ± 4,4	28,5 ± 13,5	0,036
Pressão sistólica da artéria pulmonar (mmHg)		38,3 ± 12,2	30,9 ± 10,6	<0,001
Excursão sistólica do anel tricúspide (mm)		19,2 ± 4,4	20,0 ± 4,8	0,404

*Valores são expressos como média ± desvio padrão.*

### Análise do TECP

O tratamento com sacubitril-valsartana demonstrou um impacto substancial na capacidade funcional ( Tabela 3 ). O VO_2_max, o VO_2_max previsto, a inclinação VE/VCO_2_ e a duração do exercício tiveram uma melhora importante após o tratamento, sem diferença significativa no esforço do exercício, avaliado pela razão de troca. Não foram identificadas diferenças significativas relativas aos parâmetros de frequência cardíaca (FC) e pressão arterial.

**Tabela 3:** – Dados do teste de esforço cardiopulmonar antes e seis meses depois do tratamento com sacubitril-valsartana

		Tempo 0	6 meses	p
**Dados do teste de esforço cardiopulmonar**				
Frequência cardíaca máxima (bpm)		114,1 ± 27,2	118,9 ± 24,7	0,110
Frequência cardíaca máxima prevista (%)		70,7 ± 16,0	73,9 ± 14,7	0,083
Recuperação da frequência cardíaca no primeiro minuto (bpm)		17,0 ± 12,3	17,8 ± 12,9	0,720
Pressão arterial sistólica inicial (mmHg)		115,8 ± 18,3	109,3 ± 16,5	0,094
Pressão arterial sistólica máxima (mmHg)		140,0 ± 29,8	139,7 ± 23,6	0,946
Consumo máximo de oxigênio (ml/kg/min)		14,4 ± 6,0	18,63 ± 4,9	<0,001
Consumo máximo de oxigênio previsto (%)		49,6 ± 18,7	65,7 ± 15,5	<0,001
Inclinação VE/VCO_2_		36,7 ± 7,2	31,1 ± 5,8	<0,001
Razão de troca respiratória de pico		1,0 ± 0,1	1,0 ± 0,1	0,396
Duração do exercício (s)		487,8 ± 289,3	640,3 ± 269,3	<0,001
Duração do exercício até o LA (s)		269,7 ± 277,1	292,5 ± 253,2	0,623
Consumo de oxigênio até o LA (ml/kg/min)		12,0 ± 4,3	13,7 ± 3,6	0,087

*Valores são expressos como média ± desvio padrão; LA: limiar anaeróbio.*

A Tabela 4 fornece os parâmetros do TECP de acordo com a dose de sacubitril-valsartana. Os pacientes com doses de 24/26 mg e 49/51 mg aos 6 meses de tratamento com sacubitril-valsartana apresentaram o maior aumento nos valores de VO_2_max e de inclinação VE/VCO_2_.

**Tabela 4:** – Dados do teste de esforço cardiopulmonar de acordo com a dose de sacubitril-valsartana

		Tempo 0	6 meses	p
**Dados do teste de esforço cardiopulmonar**				
**Consumo máximo de oxigênio (ml/kg/min)**				
Dose de 24/26 mg		13,5 ± 5,9	19,2 ± 6,6	0,018
Dose de 49/51 mg		13,5 ± 6,6	17,6 ± 4,3	0,019
Dose de 97/103 mg		15,5 ± 5,9	18,1 ± 4,4	0,085
**Consumo máximo de oxigênio previsto (%)**				
Dose de 24/26 mg		44,9 ± 20,6	62,8 ± 18,3	0,004
Dose de 49/51 mg		47,8 ± 18,6	69,1 ± 14,1	0,008
Dose de 97/103 mg		53,7 ± 18,2	65,2 ± 15,6	0,048
**Inclinação VE/VCO_2_**				
Dose de 24/26 mg		38,0 ± 8,9	28,1 ± 3,1	0,033
Dose de 49/51 mg		38,8 ± 5,5	31,9 ± 3,0	0,005
Dose de 97/103 mg		34,6 ± 7,3	32,0 ± 7,7	0,148

Valores são expressos como média ± desvio padrão.

Pacientes com IC tanto isquêmica (16,9 ± 7,1 ml/kg/min vs. 20,2 ± 4,2 ml/kg/min, p=0,014) quanto não-isquêmica (12,6 ± 4,6 ml/kg/min vs. 17,0 ± 5,1 ml/kg/min, p=0,004) demonstraram melhora no VO_2_max aos 6 meses de tratamento com sacubitril-valsartana.

## Discussão

O TECP é um forte preditor de mortalidade em pacientes com IC. Ele é utilizado como critério padrão para indicação de transplante cardíaco,^7^ sendo o VO_2_max e a inclinação VE/VCO_2_ os instrumentos de avaliação de risco mais utilizados.^8^ Vários tratamentos de IC (IECA, BB, AA, CDI, TRC) demonstraram melhorar a sobrevida dos pacientes e reduzir a fração de ejeção. A necessidade de revisar os critérios de priorização existentes para transplante cardíaco foi tema de debate,^15^ uma vez que o ensaio que definiu o uso de um ponto de corte ≤14 ml/kg/min para o procedimento foi publicado antes de diversos avanços no tratamento da IC.^16^

Vários ensaios com BB não foram capazes de apresentar um aumento do VO_2_max.^17 , 18^ Entretanto, o tratamento com BB aparentemente obteve um prognóstico melhor com o mesmo valor de VO_2_max,^19 , 20^ que foi utilizado para reduzir o ponto de corte para a seleção do transplante cardíaco de 14 ml/kg/min para 12 ml/kg/min.^21^ Por outro lado, a TRC apresentou aumento da capacidade de exercício em um ensaio, com crescimento médio de 1,1 ml/kg/min aos 6 meses,^22^ mas não conseguiu repetir o desempenho em outros ensaios.^23 , 24^

Com o objetivo de melhorar a capacidade funcional dos pacientes, alguns tratamentos recentes de IC, como a modulação da contratilidade cardíaca, revelaram aumento do VO_2_max de 0,65 ml/kg/min^25^ para 0,84 ml/kg/min^26^ aos 6 meses, enquanto o reparo percutâneo da insuficiência mitral secundária teve capacidade funcional preservada em um ensaio, avaliada pelo teste de caminhada de 6 minutos,^27^ mas não apresentou diferença em outro.^28^ O treinamento com exercícios também mostrou melhora no VO_2_max em ensaios anteriores, de 0,6 ml/min/kg aos 3 meses^29^ a 2,1 ml/min/kg aos 2 meses.^30^

Após o ensaio PARADIGM-HF confirmar que o tratamento com sacubitril-valsartana poderia reduzir a hospitalização por IC e a mortalidade cardiovascular em 20% em comparação com o Enalapril,^4^ o volume de informações tem aumentado, já que alguns ensaios revelaram melhora sintomática e funcional significativa depois do início do tratamento com sacubitril-valsartana.^9 - 12^ Contudo, a maioria dos ensaios tinha delineamento retrospectivo e, até onde sabemos, apenas um estudo prospectivo mostra as alterações de parâmetros do TECP após o tratamento com sacubitril-valsartana.^13^ Neste ensaio, com mediana de seguimento de 6 meses, o VO_2_max aumentou em média 2,6 ml/min/kg e a inclinação VE/VCO_2_ apresentou redução média de 2,4.

Nossos resultados mostram um grupo de pacientes com ICFER crônica altamente sintomáticos, como comprovado pela classe NYHA ≥ III em 51,4% deles (apenas 23,9% no ensaio PARADIGM-HF), pela pontuação inicial do Heart Failure Survival Score (HCSS) de 7,2 e pela taxa de hospitalização por piora da IC no ano anterior ao estudo de 42,9%. Os pacientes recebiam terapia padrão otimizada, com percentual numericamente maior de indivíduos tratados inicialmente com BB (100% vs. 93,1%), AA (94,3% vs. 52,2%), CDI (85,6% vs. 14,9%) e TRC (20% vs. 7%) em relação ao ensaio PARADIGM-HF^4^ O tratamento com sacubitril-valsartana foi iniciado com doses de 24/26 mg duas vezes ao dia em 18 (51,4%) pacientes e 49/51 mg duas vezes ao dia em 17 (48,6%) pacientes. Este procedimento é similar ao de um estudo recente de dados reais que iniciou o tratamento com sacubitril-valsartana com uma dose de 24/26 mg duas vezes ao dia em 51% dos pacientes, 49/51 mg duas vezes ao dia em 38% e 97/103 mg duas vezes ao dia em 11%.^31^ No presente estudo, a dose média diária aos seis meses foi ligeiramente maior que a do estudo anterior (251 mg/dia vs. 207 mg/dia), mas menor que a do ensaio PARADIGM-HF (375 mg/dia).^4^

Nessa população altamente sintomática, o tratamento com sacubitril-valsartana foi capaz de melhorar a classificação NYHA em pelo menos uma classe em 74,3% dos pacientes. Além da redução da classe NYHA, os dados do TECP demonstraram um aumento médio no VO_2_max de 3,9 ml/kg/min e uma redução média na inclinação VE/VCO_2_ de 5,6, um valor numericamente maior que o benefício relatado anteriormente.^13^ Os valores mais elevados da fração de ejeção do ventrículo esquerdo e do VO_2_max levaram a um crescimento significativo da HFSS (7,2 ± 1,0 vs. 7,9 ± 0,9, p=0,001).

Esses resultados podem ser importantes ao se considerar pacientes que não estão sob tratamento com sacubitril-valsartana para transplante cardíaco com base nos valores do TECP, já que aos 6 meses de tratamento, o percentual de pacientes com VO_2_max ≤12 ml/min/kg diminuiu de 37 para 11% e com inclinação VE/VCO_2_ >35, de 52,4 a 17,1%. Mais ensaios são necessários para verificar se os pontos de corte atuais para transplante cardíaco devem ser mantidos com o tratamento com sacubitril-valsartana.

Surpreendentemente, os pacientes com doses de 24/26 mg e 49/51 mg aos 6 meses de tratamento com sacubitril-valsartana apresentaram o maior aumento nos valores de VO_2_max e inclinação VE/VCO_2_, revelando o benefício desse tratamento desde que a maior dose tolerada seja administrada. Esses resultados podem complementar o contexto do uso de sacubitril-valsartana na população com ICFER, pois a dose de 24/26 mg não foi usada no ensaio PARADIGM-HF.^4^

Não é fácil explicar o aumento maior dos valores de VO_2_max e inclinação VE/VCO_2_ com a menor dose de sacubitril-valsartana. No entanto, esse cenário pode representar um viés, já que os pacientes que toleraram a maior dose de sacubitril-valsartana tinham valores iniciais de VO_2_max elevados e valores baixos de inclinação VE/VCO_2_, possivelmente tornando esse grupo menos propenso a ter um benefício maior com o tratamento.

### Limitações do estudo

Este estudo tem limitações que devem ser referenciadas na interpretação dos resultados. Esta é uma experiência prospectiva de centro único; portanto, os achados podem refletir a prática local. Embora a amostra não seja grande, o estudo mostrou resultados promissores após apenas seis meses de tratamento, o que pode ser considerado uma motivação para aumentar o uso de sacubitril-valsartana em pacientes com tal indicação, conforme recomendam as diretrizes.^5^

Apesar de ser um estudo prospectivo, foram comparados os resultados iniciais e após seis meses de tratamento com sacubitril-valsartana sem um grupo controle que continuaria o tratamento com IECA ou BRA. Após os resultados do ensaio PARADIGM-HF,^4^ não seria ético deixar alguns dos pacientes sem um tratamento que demonstrou melhorar a sobrevida.

Uma estratégia para tentar reduzir o viés relacionado à melhora concomitante causada por outros tratamentos que não o com sacubitril-valsartana foi a escolha de pacientes para o estudo previamente sob terapia padrão otimizada (exceto o tratamento com sacubitril-valsartana) por mais de seis meses e que passaram por procedimento cardiovascular significativo não recente (implante de CDI ou TRC, procedimento de revascularização coronariana, tratamento valvar ou ablação por cateter de fibrilação atrial). Isso fica evidente por não haver diferenças na dosagem de BB e AA após seis meses de tratamento ou novo procedimento de revascularização coronariana, tratamento valvar ou ablação por cateter de fibrilação atrial.

## Conclusões

O tratamento com sacubitril-valsartana aparentemente aumentou a capacidade funcional de pacientes com IC crônica, com melhora acentuada no VO_2_max, VO_2_max previsto, inclinação VE/VCO_2_ e duração do exercício, bem como redução da classe NYHA. Esses resultados podem complementar o contexto do uso de sacubitril-valsartana na população com ICFER, mostrando manutenção do benefício mesmo com a menor dose do tratamento, desde que esta seja a maior dose tolerada.

## *Material suplementar

Para informação adicional, por favor,clique aqui


