# Supplementary material for: Melhora no Consumo Máximo de Oxigênio e na Ventilação após Tratamento com Sacubitril-Valsartana
Source: Arq Bras Cardiol. 2020 Oct 9;115(5):821–7. [Article in Portuguese] doi: 10.36660/abc.20190443 (PMC8452225; doi:10.36660/abc.20190443)
Supplement: Supplementary file 1 [file 2019-0443-supplementary-appendix.pdf]

SUPPLEMENTARY APPENDIX

Collected data
**CHARACTERISTICS**
Age
Etiology
Gender
NYHA class
Body mass index
Heart failure hospitalization in the previous year
Smokers
Previous hypertension
Dyslipidemia
Diabetes mellitus
Peripheral artery disease
Familiar history of Heart Failure
Atrial Fibrillation
Chronic kidney disease
Chronic liver disease
Angiotensin-converting enzyme inhibitors
Angiotensin II receptor blocker
Beta-blockers
Mineralocorticoid receptor antagonist
Ivabradine
Digoxin
Implantable cardioverter defibrillator
Cardiac resynchronization therapy
Percutaneous mitral-valve repair using a MitraClip®

Collected data
**OUTCOMES**
Death
Cardiac death
Sudden cardiac death
Non-cardiac death
Heart failure hospitalization
Need for inotropic support
Heart transplantation
Left ventricular assist device
Adverse events requiring discontinuation

Collected data
**HEART FAILURE PROGNOSTIC SCORES**
Heart Failure Survival Score
Seattle Heart Failure Model (1 year expected survival)
Seattle Heart Failure Model (5 years expected survival)
MAGGIC score
1 year MAGGIC score risk of dying
3 years MAGGIC score risk of dying

Collected data
**LABORATORIAL DATA**
Hemoglobin (mg/dL)
Mean corpuscular volume (fL)
Ferritin (ng/mL)
INR
Total bilirubin (mg/dL)
Aspartate transaminase (U/L)
Alanine transaminase (U/L)
Glomerular filtration rate (ml/min)
Creatinine (mg/dL)
Urea (mg/dL)
Potassium (mEq/L)
Sodium (mEq/L)
Creatine Kinase (U/L)
Uric acid (mg/dL)
Glycated hemoglobin (%)
Thyroid-stimulating hormone (mU/L)
Creatine Kinase (U/L)
Troponin I (pg/ml)
NT-proBNP (pg/ml)
BNP (pg/ml)

Collected data
**ELECTROCARDIOGRAPHIC DATA**
Heart rate (bpm)
PQ interval (msec)
QRS interval (msec)
QTc interval (msec)
SV2 + RV5 (mm)
Biventricular pacing (%)
Cardiac rhythm
Intraventricular conduction block
Presence of Q waves

Collected data
**CARDIOPULMONARY EXERCISE TEST DATA**
Maximal heart rate (bpm)
Maximal predicted heart rate (%)
First minute heart rate recovery (bpm)
Initial systolic blood pressure (mmHg)
Maximal systolic blood pressure (mmHg)
Peak oxygen consumption (ml/kg/min)
Peak predicted oxygen consumption (%)
VE/VCO_2 slope
Peak ratio exchange ratio
Duration of exercise (sec)
Duration of exercise until anaerobic threshold (sec)
Oxygen consumption at anaerobic threshold (ml/kg/min)

Collected data
**ECHOCARDIOGRAPHIC DATA**
Left ventricle end-diastolic diameter (mm)
Left ventricle end-systolic diameter (mm)
Interventricular septum (mm)
Left ventricular ejection fraction (%)
Global longitudinal strain (%)
Myocardial Constructive Work (mmHg)
Myocardial Wasted Work (mmHg)
Myocardial work index (mmHg%)
Myocardial Work Efficiency (%)
E/e´
Pulmonary artery systolic pressure (mmHg)
Left atrium volume (ml/m^2)
Right atrium volume (ml/m^2)
Mechanical dispersion (ms)
Diastolic dysfunction grade
Mitral regurgitation grade
Tricuspid regurgitation grade
Aortic regurgitation grade
E (cm/s)
A (cm/s)
Mean e´ (cm/s)
Tricuspid regurgitant velocity (m/sc)
Presence of pericardial effusion
Inferior vena cava index
