# Supplementary material for: Melhora no Consumo Máximo de Oxigênio e na Ventilação após Tratamento com Sacubitril-Valsartana
Source: Arq Bras Cardiol. 2020 Oct 9;115(5):821–7. [Article in Portuguese] doi: 10.36660/abc.20190443 (PMC8452225; doi:10.36660/abc.20190443)
Supplement: Translation

# Translation: Maximal Oxygen Uptake and Ventilation Improvement Following Sacubitril-Valsartan Therapy

**Authors:** António Valentim Gonçalves, Tiago Pereira-da-Silva, Ana Galrinho, Pedro Rio, Rui Soares, Joana Feliciano, Rita Ilhão Moreira, Sofia Silva, Sandra Alves, Eunice Capilé, Rui Cruz Ferreira

**Affiliations:** 1 Portugal Hospital de Santa Marta, Lisboa – Portugal

**Keywords:** Heart Failure, Oxygen Consumption, Stroke Volume, Pulmonary Ventilation, Sacubitril-Valsartan, Hypertension, Antihypertensive Agents

## Abstract

**Background:**

Sacubitril/valsartan had its prognosis benefit confirmed in the PARADIGM-HF trial. However, data on cardiopulmonary exercise testing (CPET) changes with sacubitril-valsartan therapy are scarce.

**Objective:**

This study aimed to compare CPET parameters before and after sacubitril-valsartan therapy.

**Methods:**

Prospective evaluation of chronic heart failure (HF) patients with left ventricular ejection fraction ≤40% despite optimized standard of care therapy, who started sacubitril-valsartan therapy, expecting no additional HF treatment. CPET data were gathered in the week before and 6 months after sacubitril-valsartan therapy. Statistical differences with a p-value <0.05 were considered significant.

**Results:**

Out of 42 patients, 35 (83.3%) completed the 6-month follow-up, since 2 (4.8%) patients died and 5 (11.9%) discontinued treatment for adverse events. Mean age was 58.6±11.1 years. New York Heart Association class improved in 26 (74.3%) patients. Maximal oxygen uptake (VO_2_max) (14.4 vs. 18.3 ml/kg/min, p<0.001), VE/VCO_2_slope (36.7 vs. 31.1, p<0.001), and exercise duration (487.8 vs. 640.3 sec, p<0.001) also improved with sacubitril-valsartan. Benefit was maintained even with the 24/26 mg dose (13.5 vs. 19.2 ml/kg/min, p=0.018) of sacubitril-valsartan, as long as this was the highest tolerated dose.

**Conclusions:**

Sacubitril-valsartan therapy is associated with marked CPET improvement in VO_2_max, VE/VCO_2_slope, and exercise duration. (Arq Bras Cardiol. 2020; [online].ahead print, PP.0-0)

## Introduction

The prognosis of heart failure (HF) patients remarkably changed following the publication of cornerstone trials (1987 – CONSENSUS,^1^ 1999 – CIBIS-II,^2^ and RALES^3^ ), which demonstrated the benefit of using neurohormonal antagonists [angiotensin-converting enzyme inhibitors (ACEI), beta-blockers (BB), and mineralocorticoid receptor antagonist (MRA), respectively] to improve patient survival and reduce ejection fraction.

Twenty-seven years after the CONSENSUS trial, the PARADIGM-HF trial showed that sacubitril-valsartan, a combination of neprilysin inhibitor and angiotensin II receptor blocker (ARB), could reduce both HF hospitalization and cardiovascular mortality in 20% in comparison with Enalapril.^4^

As a result, sacubitril-valsartan has a Class I recommendation, level of evidence B, as a replacement for ACEI to ambulatory patients with HF with reduced ejection fraction (HFrEF) who remain symptomatic despite optimal treatment with ACEI (or ARB if ACEI is not tolerated), BB, and MRA.^5^ However, the use of sacubitril-valsartan has not been as high as expected.^6^

The treatment goals for HF patients are not only to prevent hospital admission and reduce mortality but also to improve their clinical status and functional capacity. Cardiopulmonary exercise testing (CPET) is a powerful predictor of mortality in HF patients. It is considered the standard criterion for evaluating the need for elective heart transplantation,^7^ with maximal O_2_uptake (VO_2_max) and the relationship between ventilation and CO_2_production (VE/VCO_2_slope) as the most adopted risk assessment tools.^8^

Information has been increasing recently, as some trials demonstrated a significant symptomatic and functional improvement following the initiation of sacubitril-valsartan therapy.^9 - 12^ Nonetheless, its impact on functional capacity needs additional research since most trials had retrospective designs, and, to the best of our knowledge, only one prospective study shows CPET parameter changes after sacubitril-valsartan therapy.^13^

This study aimed to prospectively analyze the effectiveness of sacubitril-valsartan therapy in a cohort of chronic HF patients with optimized standard of care therapy by comparing CPET data before and after treatment.

## Methods

The investigation conforms to the principles outlined in the Declaration of Helsinki. The institutional ethics committee and the National Commission for Data Protection ( *Comissão Nacional de Proteção de Dados* – CNPD, authorization number 5962) approved the study protocol.

All patients provided written informed consent.

### Patient population

The study included a prospective single-center analysis from October 2017 to June 2018.

During this period, all ambulatory patients with optimized standard of care therapy for chronic HF, left ventricular ejection fraction ≤40%, and New York Heart Association (NYHA) class ≥ II were advised to start sacubitril-valsartan therapy according to the current guidelines.^5^

### Definition of chronic HF with optimized standard of care therapy

Optimized standard of care therapy for chronic HF was defined as more than six months of treatment with the maximum tolerated dose of an ACEI or ARB, as appropriate, a BB, and an MRA. Implantable cardioverter-defibrillator (ICD) and/or cardiac resynchronization therapy (CRT) can be used if indicated by the current guidelines and if the subject has been adequately treated per applicable standards for coronary artery disease and mitral regurgitation (MR)^5^ and no additional HF treatment was expected to change in the next 6 months. Patients who started an exercise program in the three months previous to or during sacubitril-valsartan therapy were excluded.

### Study protocol

All patients provided written informed consent. Thereafter, clinical, laboratory, transthoracic echocardiography (TTE), and CPET data were obtained in the week before starting sacubitril-valsartan therapy.

A washout period of 36 hours allowed switching from an ACEI to sacubitril-valsartan. Sacubitril-valsartan therapy was preferentially started at 49/51 mg twice daily or 24/26 mg twice daily in patients with a dose <10 mg/day of Enalapril or equivalent. Attempts to double the dose were made every 2 to 4 weeks to reach the target maintenance dose of 97/103 mg twice daily, except in patients with systolic blood pressure <100 mmHg, symptomatic hypotension, hyperkalemia > 5.5 mEq/L, or a decrease in glomerular filtration rate (GFR) to less than 60 ml/min, as assessed by the Cockcroft-Gault equation.

All patients were followed for six months from the test completion date, and clinical, laboratory, TTE, and CPET data were collected again after six months of sacubitril-valsartan therapy.

The supplementary appendix provides information regarding all data collected.

### Cardiopulmonary exercise testing

A maximal symptom-limited treadmill CPET was performed using the modified Bruce protocol (GE Marquette Series 2000 treadmill). Gas analysis was preceded by calibration of the equipment. Minute ventilation, oxygen uptake, and carbon dioxide production were acquired breath-by-breath, using a SensorMedics Vmax 229 gas analyzer. The VO_2_max was defined as the highest 30-second average achieved during exercise and was normalized for body mass index.^14^ Anaerobic threshold (AT) was determined by combining the standard methods (V-slope preferentially and ventilatory equivalents). VE/VCO_2_slope was calculated by least-squares linear regression, based on data acquired throughout the whole exercise. Patients were encouraged to perform the exercise until the respiratory exchange ratio (RER) was ≥1.10.

### Statistical analysis

Baseline characteristics are expressed as frequencies (percentages) for categorical variables and as means and standard deviations for continuous variables. All analyses compare patients’ parameters at baseline and after six months of sacubitril-valsartan therapy.

Normal distribution of continuous variables was verified by the Kolmogorov-Smirnov test. Paired samples *t* -test compared the variables before and after sacubitril-valsartan therapy. Statistical differences with a p-value <0.05 were considered significant. Data were analyzed in the software Statistical Package for the Social Science for Windows, version 24.0 (SPSS Inc, Chicago, IL).

## Results

### Overview of the study population

A total of 42 patients were enrolled in the study. Out of them, 35 (83.3%) completed the six-month follow-up with sacubitril-valsartan, since 2 (4.8%) patients died (1 with intracranial hemorrhage after trauma and 1 with sudden cardiac death) and 5 (11.9%) patients discontinued treatment due to adverse events (2 with reversible acute kidney injury and 3 with symptomatic hypotension with the lowest sacubitril-valsartan dose). No patient was lost to follow-up during the six months.

Table 1 presents the baseline characteristics of the 35 patients who completed the six-month follow-up with sacubitril-valsartan. Mean age was 58.6±11.1 years, with 29 (82.9%) male patients and ischemic etiology in 15 (42.9%) participants.

**Table 1:** – Baseline characteristics of the study population (n=35)

Characteristics	n (%)
Mean age (years)	58.60 ± 11.14
Ischemic etiology	15 (42.9%)
Male gender	29 (82.9%)
NYHA ≥ III	18 (51.4%)
Mean body mass index (kg/m^2^)	28.09 ± 3.77
Heart failure hospitalization in the previous year	15 (42.9%)
Mean BNP (pg/ml)	375.30 ± 342.19
Current smoker	7 (20.0%)
Previous hypertension	25 (71.4%)
Dyslipidemia	25 (71.4%)
Diabetes mellitus	11 (31.4%)
Peripheral arterial disease	4 (11.4%)
Family history of heart failure	1 (2.9%)
Atrial fibrillation	14 (40%)
Chronic kidney disease	2 (5.7%)
Chronic liver disease	0 (0.0%)
Angiotensin-converting enzyme inhibitors	29 (82.9%)
Angiotensin II receptor blocker	6 (17.1%)
Beta-blockers	35 (100.0%)
Mineralocorticoid receptor antagonist	33 (94.3%)
Ivabradine	13 (37.1%)
Digoxin	9 (25.7%)
Implantable cardioverter-defibrillator	30 (85.6%)
Cardiac resynchronization therapy (CRT-D)	7 (20%)
Percutaneous mitral valve repair using MitraClip®	3 (8.6%)

*NYHA: New York Heart Association; BNP: B-type natriuretic peptide.*

These patients were highly symptomatic, as revealed by an NYHA class ≥ III in 51.4% of them and by 42.9% of hospitalizations for worsening HF in the year prior to sacubitril-valsartan therapy. All patients were on ACEI or ARB associated with a BB, and 94.3% were taking an MRA. ICD was already implanted in 30 (85.6%) patients, out of which 7 (20.0%) had a CRT-D system. Three (8.6%) patients had formerly undergone percutaneous mitral valve repair using a MitraClip® system.

### Sacubitril-valsartan dose

Sacubitril-valsartan therapy was started at 24/26 mg twice daily in 18 (51.4%) patients and 49/51 mg twice daily in 17 (48.6%) patients. At six months, a dose of 24/26 mg twice daily was administered to 10 (28.6%) patients, 49/51 mg twice daily to 11 (31.4%), and 97/103 mg twice daily to 14 (40.0%).

We found no significant changes regarding the dose expressed as a percentage of the target dose of BB (68.8 ± 28.6% vs. 70.6 ± 28.0%, p=0.278) and MRA (51.6 ± 19.0% vs. 53.2 ± 24.4%, p=0.352) or the loop diuretic dose expressed as furosemide equivalents (43.6 ± 27.6% vs. 39.1 ± 26.5%, p=0.191) at baseline and after six months of sacubitril-valsartan therapy.

### Clinical assessment

The 35 patients who completed six months of sacubitril-valsartan treatment showed a relevant improvement in NYHA class, since only 9 (25.7%) patients remained in the same class, while 24 (68.6%) improved one NYHA class and 2 (5.7%) improved two classes. No patient had a worsening in their NYHA class during the six months of sacubitril-valsartan therapy, and only 3 (8.6%) remained in class III.

### Transthoracic echocardiography assessment

Table 2 presents the results of the TTE analysis. Left ventricular (LV) dimensions and atrial volumes were significantly lower at six months of treatment. Tricuspid annular plane systolic excursion showed no significant differences, regardless of the presence of a decrease in pulmonary artery systolic pressure at 6 months of therapy. Left ventricular ejection fraction had a mean absolute raise of 5.9%.

**Table 2:** – Echocardiographic data before and after six months of sacubitril-valsartan therapy

		Time 0	6 months	p
ECHOCARDIOGRAPHIC DATA				
Left ventricular end-diastolic diameter (mm)		71.3 ± 8.4	66.9 ± 7.6	0.001
Left ventricular end-systolic diameter (mm)		57.8 ± 9.4	53.1 ± 9.3	0.002
Interventricular septum (mm)		9.6 ± 1.7	9.9 ± 1.9	0.280
Left ventricular ejection fraction (%)		29.3 ± 6.4	35.17 ± 8.6	0.001
Left atrial volume (ml/m^2^)		51.5 ± 22.6	43.7 ± 15.8	0.004
Right atrial volume (ml/m^2^)		33.1 ± 4.4	28.5 ± 13.5	0.036
Pulmonary artery systolic pressure (mmHg)		38.3 ± 12.2	30.9 ± 10.6	<0.001
Tricuspid annular plane systolic excursion (mm)		19.2 ± 4.4	20.0 ± 4.8	0.404

*Values are expressed as mean ± standard deviation.*

### CPET analysis

Sacubitril-valsartan therapy showed a remarkable impact on functional capacity ( Table 3 ). The VO_2_max, predicted VO_2_max, VE/VCO_2_slope, and duration of exercise presented an important improvement after therapy, without a significant difference in exercise effort, as assessed by the exchange ratio. We found no significant differences regarding heart rate (HR) and blood pressure parameters.

**Table 3:** – Cardiopulmonary exercise testing data before and after six months of sacubitril-valsartan therapy

		Time 0	6 months	p
**Cardiopulmonary exercise testing data**				
Maximal heart rate (bpm)		114.1 ± 27.2	118.9 ± 24.7	0.110
Maximal predicted heart rate (%)		70.7 ± 16.0	73.9 ± 14.7	0.083
One-minute heart rate recovery (bpm)		17.0 ± 12.3	17.8 ± 12.9	0.720
Initial systolic blood pressure (mmHg)		115.8 ± 18.3	109.3 ± 16.5	0.094
Maximal systolic blood pressure (mmHg)		140.0 ± 29.8	139.7 ± 23.6	0.946
Maximal oxygen uptake (ml/kg/min)		14.4 ± 6.0	18.63 ± 4.9	<0.001
Predicted maximal oxygen uptake (%)		49.6 ± 18.7	65.7 ± 15.5	<0.001
VE/VCO_2_slope		36.7 ± 7.2	31.1 ± 5.8	<0.001
Peak respiratory exchange ratio		1.0 ± 0.1	1.0 ± 0.1	0.396
Duration of exercise (sec)		487.8 ± 289.3	640.3 ± 269.3	<0.001
Duration of exercise until AT (sec)		269.7 ± 277.1	292.5 ± 253.2	0.623
Oxygen uptake at AT (ml/kg/min)		12.0 ± 4.3	13.7 ± 3.6	0.087

*Values are expressed as mean ± standard deviation; AT: anaerobic threshold.*

Table 4 provides CPET parameters by sacubitril-valsartan dose. Patients on 24/26 mg and 49/51 mg doses at 6 months of sacubitril-valsartan therapy had the highest increase in VO_2_max and VE/VCO_2_slope values.

**Table 4:** – Cardiopulmonary exercise testing data by sacubitril-valsartan dose

		Time 0	6 months	p
**Cardiopulmonary exercise testing data**				
**Maximal oxygen uptake (ml/kg/min)**				
24/26 mg dose		13.5 ± 5.9	19.2 ± 6.6	0.018
49/51 mg dose		13.5 ± 6.6	17.6 ± 4.3	0.019
97/103 mg dose		15.5 ± 5.9	18.1 ± 4.4	0.085
**Predicted maximal oxygen uptake (%)**				
24/26 mg dose		44.9 ± 20.6	62.8 ± 18.3	0.004
49/51 mg dose		47.8 ± 18.6	69.1 ± 14.1	0.008
97/103 mg dose		53.7 ± 18.2	65.2 ± 15.6	0.048
**VE/VCO_2_slope**				
24/26 mg dose		38.0 ± 8.9	28.1 ± 3.1	0.033
49/51 mg dose		38.8 ± 5.5	31.9 ± 3.0	0.005
97/103 mg dose		34.6 ± 7.3	32.0 ± 7.7	0.148

*Values are expressed as mean ± standard deviation.*

Both ischemic (16.9 ± 7.1 ml/kg/min vs. 20.2 ± 4.2 ml/kg/min, p=0.014) and non-ischemic (12.6 ± 4.6 ml/kg/min vs. 17.0 ± 5.1 ml/kg/min, p=0.004) HF patients showed VO_2_max improvement at 6 months of sacubitril-valsartan therapy.

## Discussion

CPET is a powerful predictor of mortality in HF patients. It is considered the standard indication criterion for heart transplantation,^7^ with VO_2_max and VE/VCO_2_slope as the most adopted risk assessment tools.^8^ Several HF treatments (ACEI, BB, MRA, ICD, CRT) have proven to improve patient survival and reduce ejection fraction. Whether to revise the existing listing criteria for heart transplantation was a matter of debate,^15^ since the trial that defined the use of a cut-off point ≤14 ml/kg/min for the procedure was published before several advancements in HF treatment.^16^

Several trials with BB failed to demonstrate an increase in VO_2_max.^17 , 18^ However, BB therapy seemed to provide a better prognosis with the same VO_2_max value,^19 , 20^ which was used to reduce the cut-off point for heart transplantation selection from 14 ml/kg/min to 12 ml/kg/min.^21^ On the other hand, CRT showed an increase in exercise capacity in one trial, with a mean growth of 1.1 ml/kg/min at 6 months,^22^ but failed to do the same in other trials.^23 , 24^

Aiming at improving patients’ functional capacity, some recent HF treatments, like cardiac contractility modulation, revealed an improvement in VO_2_max from 0.65 ml/kg/min^25^ to 0.84 ml/kg/min^26^ at 6 months, while percutaneous repair for secondary mitral regurgitation had preserved functional capacity in one trial, as assessed by the 6-minute walk test,^27^ but no differences in another.^28^ Exercise training has also shown a VO_2_max improvement in previous trials, from 0.6 ml/min/kg at 3 months^29^ to 2.1 ml/min/kg at 2 months.^30^

After the PARADIGM-HF trial confirmed that sacubitril-valsartan therapy could reduce both HF hospitalization and cardiovascular mortality by 20% in comparison with Enalapril,^4^ information has been increasing, as some trials revealed a significant symptomatic and functional improvement following the initiation of sacubitril-valsartan therapy.^9 - 12^ Nevertheless, most trials had retrospective designs, and, to the best of our knowledge, only one prospective study shows CPET parameter changes after sacubitril-valsartan therapy.^13^ In this trial, with a median follow-up of 6 months, VO_2_max increased by 2.6 ml/min/kg on average, and VE/VCO_2_slope had a mean reduction of 2.4.

Our results show a group of highly symptomatic chronic HFrEF patients, as revealed by an NYHA class ≥ III in 51.4% of them (only 23.9% in the PARADIGM-HF trial), a baseline Heart Failure Survival Score (HFSS) of 7.2, and a hospitalizations rate for worsening HF in the year prior to the study of 42.9%. Patients were on optimized standard of care therapy, with a numerically higher percentage of individuals treated at baseline with BB (100% vs. 93.1%), MRA (94.3% vs. 52.2%), ICD (85.6% vs. 14.9%), and CRT (20% vs. 7%) when compared to the PARADIGM-HF trial.^4^ Sacubitril-valsartan therapy was started at 24/26 mg twice daily in 18 (51.4%) patients and 49/51 mg twice daily in 17 (48.6%) patients. This procedure is in line with a recent real-world data study that started sacubitril-valsartan therapy at 24/26 mg twice daily in 51% of patients, 49/51 mg twice daily in 38%, and 97/103 mg twice daily in 11%.^31^ In our trial, the mean daily dose at six months was slightly higher than the previous trial (251 mg/day vs. 207 mg/day) but lower than the PARADIGM-HF trial (375 mg/day).^4^

In this highly symptomatic population, sacubitril-valsartan therapy was able to improve the NYHA classification by at least one class in 74.3% of patients. In addition to the reduction in NYHA class, CPET data demonstrated a mean VO_2_max increase of 3.9 ml/kg/min and a mean VE/VCO_2_slope decrease of 5.6, which is numerically higher than the benefit previously reported.^13^ Higher values of left ventricular ejection fraction and VO_2_max led to significant HFSS growth (7.2 ± 1.0 vs. 7.9 ± 0.9, p=0.001).

These results could be important when considering patients not on sacubitril-valsartan therapy for heart transplantation based on CPET values, since at 6 months of treatment, the percentage of patients with VO_2_max ≤12 mL/min/kg decreased from 37 to 11% and with VE/VCO_2_slope >35, from 52.4 to 17.1%. Further trials are necessary to verify whether the current cut-off points for heart transplantation should be maintained with sacubitril-valsartan therapy.

Surprisingly, patients receiving 24/26 mg and 49/51 mg doses at 6 months of sacubitril-valsartan therapy had the highest increase in VO_2_max and VE/VCO_2_slope values, revealing the benefit of this treatment as long as the highest tolerated dose was administered. These results can complement the background of sacubitril-valsartan use in the HFrEF population, since the 24/26 mg dose was not used in PARADIGM-HF trial.^4^

The highest increase in VO_2_max and VE/VCO_2_slope values with the lowest sacubitril-valsartan dose is not easy to explain. Nonetheless, this scenario could represent a bias since patients who tolerated the highest dose of sacubitril-valsartan had high VO_2_max baseline values and small VE/VCO_2_slope values, possibly making this group less prone to a greater benefit with the therapy.

### Study limitations

Our study has limitations that should be referenced when interpreting the results. This is a single-center prospective experience; therefore, the findings might reflect local practice. Although the sample was not large, the study showed promising results after only six months of therapy, which can be considered a motivation to increase the use of sacubitril-valsartan in patients with such indication, as recommended by the guidelines.^5^

Despite being a prospective study, the results were compared between baseline and after six months of sacubitril-valsartan therapy without a control group that would continue ACEI or ARB therapy. After the results of the PARADIGM-HF trial,^4^ leaving some patients without a therapy that has proven to improve survival would not be ethical.

A strategy to try to reduce bias related to concomitant improvement caused by therapies other than sacubitril-valsartan was choosing study patients with previous optimized standard of care therapy (except for sacubitril-valsartan therapy) for more than six months and non-recent major cardiovascular procedure (ICD or CRT implantation, coronary revascularization procedure, valvular treatment, or catheter ablation of atrial fibrillation). This is demonstrated by the lack of differences in BB and MRA dosage after six months of therapy and because no new coronary revascularization procedure, valvular treatment, or catheter ablation of atrial fibrillation was performed.

## Conclusions

Sacubitril-valsartan therapy seemed to increase the functional capacity of chronic HF patients, with a marked improvement in VO_2_max, predicted VO_2_max, VE/VCO_2_slope, and duration of exercise, as well as a reduction in NYHA class. These results can complement the background of sacubitril-valsartan use in the HFrEF population, showing benefit even with the lowest dose of therapy as long as this was the highest tolerated dose.

## * Supplemental Materials

For additional information, please click here.
